# Evaluating the effect of fucoidan-alginate combined dressing on wound healing in rats with full-thickness skin removed

**DOI:** 10.22038/ijbms.2025.86338.18650

**Published:** 2025

**Authors:** Guifa Wang, Nan Zhang, Xiaochen Zhang, Zihan Guo, Man Liu, Meilan Xue, Hui Liang

**Affiliations:** 1 Department of Biochemistry and Molecular Biology, School of Basic Medicine, Qingdao University, 308 Ningxia Road, Qingdao, 266071, China; 2 Department of Nutrition and Food Hygiene, School of Public Health, Qingdao University, 308 Ningxia Road, Qingdao, 266071, China; 3 Adelaide 128 Waymouth St, Y Suites on Waymouth Adelaide SA 5000 Australia; 4 Department of Clinical Nutrition, The Second People’s Hospital of Lianyungang, Lianyungang, 222000, China; 5 School of Nursing, Qingdao University, 308 Ningxia Road, Qingdao, 266071, China

**Keywords:** Alginate, Combined dressing Fucoidan, Natural product, Wound healing

## Abstract

**Objective(s):**

This study aims to investigate the effects of fucoidan-alginate combined dressings on wound healing in rats with full-thickness skin defects and to explore the underlying mechanisms.

**Materials and Methods:**

Male SD rats were divided into three groups (n=15): Control, 2% fucoidan, and 5% fucoidan. Full-thickness skin wounds were created on each rat. Fucoidan-alginate dressings were prepared by applying 20 mg/ml and 50 mg/ml fucoidan solutions to alginate dressings (2×2 cm), resulting in 2% and 5% (w/v) fucoidan-alginate combined dressings, respectively. The control group utilized alginate dressings. Wound healing was assessed through various methods, including wound area measurement, histopathological analysis, white blood cell counts, ELISA for TNF-α and IL-1β, Masson’s trichrome staining for collagen, immunohistochemistry for TGF-β1, and western blotting for TGF-β1 and Smad-related proteins.

**Results:**

The results revealed that wound healing was significantly more effective in rats treated with 5% fucoidan-alginate combined dressings. Compared to the control group (*P<*0.01) and the 2% FUC group (*P<*0.05), the 5% FUC group exhibited reduced inflammatory cell infiltration and lower levels of TNF-α and IL-1β. Moreover, in comparison to the control group, the 5% FUC group demonstrated a significant up-regulation in the mean density of TGF-β1 (*P<*0.01) and significantly elevated protein expression levels of Col I, α-SMA, and p-Smad2/3 (*P<*0.01). Additionally, a notable amount of collagen production was observed.

**Conclusion:**

The findings suggested that fucoidan-alginate dressings promote wound healing, reduce inflammation, and enhance collagen synthesis in rats, likely via the TGF-β1/Smad signaling pathway.

## Introduction

Wounds, typically caused by external forces or underlying diseases, refer to the disruption of skin integrity, often resulting in tissue loss and, in severe cases, functional impairment (1). Based on their healing timelines, wounds are categorized into two types: acute wounds (such as surgical wounds, trauma, and burns) and chronic wounds (such as varicose ulcers, diabetic foot ulcers, and pressure ulcers) (2). Acute wounds, when complicated by extensive necrosis or severe infection, can lead to amputation or even become life-threatening, significantly impacting health and quality of life (3). Chronic wounds, often associated with other chronic conditions, have been described as a “silent epidemic” affecting a significant portion of the global population (4). The growing burden of chronic non-communicable diseases (NCDs)-including diabetes, vascular diseases, obesity, and cancer—due to population aging has become a major driver behind the prevalence of chronic wounds (5-7). Moreover, individuals with wounds frequently experience pain, functional impairment, diminished productivity, social isolation, and extended hospital stays. These challenges are often compounded by psychological issues, such as depression, distress, and anxiety (8). As a result, wounds represent an urgent global public health challenge, placing a substantial burden on healthcare systems worldwide.

Wound healing, a complex and highly regulated physiological process, relies on the coordinated actions of various cells and mediators to restore injured skin to its normal barrier function (9, 10). This process can be broadly divided into four distinct stages: hemostasis, inflammation, proliferation, and remodeling. These stages are intricately linked to several biological events, including epithelial cell migration, inflammatory cell infiltration, granulation tissue formation, and collagen deposition (5, 11). A key research focus in the field of wound healing is the transition from the inflammatory stage to the proliferative stage. The shift from M1 macrophages to M2 macrophages serves as an early indicator of progression toward proliferation. Upon entering the proliferative stage, M2 macrophages release TGF-β1, initiating granulation tissue formation and promoting the proliferation of fibroblasts (12). The TGF-β1/Smad signaling pathway, a pivotal mechanism for collagen production, plays a critical role in epithelial-mesenchymal transformation (EMT). This pathway stimulates fibroblasts to differentiate into myofibroblasts, enhances the deposition of extracellular matrix (ECM) proteins, and is indispensable for the contraction of granulation tissue (13-15). In recent years, alginate dressings have garnered significant attention in the field of wound care due to their unique properties. Primarily composed of calcium alginate derived from brown seaweed, these dressings are widely utilized in clinical practice for effective wound management (16). They exhibit excellent absorbency, capable of soaking up substantial amounts of exudate from wounds, thereby maintaining a moist environment that is conducive to healing (17). However, the healing of complex wounds often necessitates more potent promoting effects. Consequently, researchers have started to investigate the combination of alginate dressings with other bioactive substances to further enhance their wound-healing properties.

Derived from brown algae and marine invertebrates, fucoidan is primarily composed of fucose and sulfate groups, with small amounts of mannose, galactose, and uronic acid. It has been widely recognized for its extensive pharmacological effects (18, 19). Numerous studies have clearly demonstrated that fucoidan exhibits a broad range of biological activities, both in vitro and in vivo, including anti-inflammatory, antiviral, immunomodulatory, and anticancer effects (19, 20). Park *et al*. revealed that the topical application of low molecular weight fucoidan (LMWF) extracted from* Undaria pinnatifida* significantly promoted wound contraction. The underlying mechanism was primarily associated with the increased expression of TGF-β1 and VEGFR2, which enhanced fibroblast regeneration and angiogenesis (21). Additionally, the antioxidant properties of fucoidan help reduce oxidative stress, thereby alleviating damage to tissues and cellular structures caused by oxidative damage (21, 22). Moreover, as a marine polysaccharide, fucoidan possesses moisturizing properties, creating a moist environment conducive to wound healing (23-25). Based on the excellent wound-healing properties of alginate dressings and the biological activities of fucoidan, we speculated that combining fucoidan with alginate dressings might create a more powerful synergistic effect for promoting wound healing. In this study, a rat full-thickness skin wound model was established to evaluate the wound healing potential of fucoidan-alginate combined dressings and investigate their related mechanisms.

## Materials and Methods

### Materials

Both the fucoidan and the alginate dressing were provided by Bright Moon Seaweed Group Co., Ltd (Qingdao, China). The molecular weight of fucoidan (90% purity) is about 220~260 KD, and the content of organic SO4 ^2-^ is 29.65%. The main component of alginate dressings is calcium alginate (90% purity), of which the CaO content is 8~12%. H&E staining kit, Masson staining kit, and DAB color development solution were purchased from Beijing Solarbio Science & Technology Co., Ltd (Beijing, China). Primary antibodies for Interleukin-1β (IL-1β), tumor necrosis factor-α (TNF-α), and transforming growth factor-β1 (TGF-β1) were obtained from Santa Cruz Biotechnology, Inc. (Shanghai, China). Antibodies for α-smooth muscle actin (α-SMA), collagen Type I (Col I), collagen Type III (Col III), and β-actin were obtained from Chengdu Zen-Bioscience Co., Ltd. (Chengdu, China). Antibodies for p-Smad/2/3 were obtained from ABclonal Biotechnology Co., Ltd. (Wuhan, China). Anti-Rabbit IgG H&L (HRP) and Alexa Fluor™ 488 secondary antibodies were purchased from Abcam plc (Cambridge, UK). The IL-1 β, TNF-α, and TGF-β 1 ELISA kits are provided by Beyotime Biotech Inc (Shanghai, China).

### Dressing preparation

Fucoidan powder was dissolved in normal saline and diluted into 20 mg/ml (w/v) and 50 mg/ml (w/v) solutions, respectively. The semi-moist 2% (w/v) fucoidan dressing was made by dropping 50 μl (w/v) of 20 mg/ml (w/v) fucoidan solution onto the alginate dressing (size 2×2 cm square). Moreover, the semi-moist 5% (w/v) fucoidan-alginate combined dressing was prepared as above. The control dressing was made by dropping 50 μl of normal saline onto the alginate dressing.

### Animal treatment and intervention strategy

Forty-five male Sprague-Dawley (SD) rats (7 weeks old, weight 200±20 g) were provided by Pengyue Experimental Animal Breeding Co., Ltd (Jinan, China). All experimental procedures in this study were approved by the Review Committee for the Use of Human or Animal Subjects of the Department of Medicine, Qingdao University, and were performed following the U.K. Animals (Scientific Procedures) Act, 1986, and the relevant guidelines for animal experiments. 

Each rat was kept in a single cage in an air-conditioned room with constant temperature (22±1 ^°^C), constant humidity (50~60%), and light-dark cycle of 12h for one week of adaptive feeding. The rats were then numbered and randomly divided into the following three groups (15 rats/group): Control group (Control), 2% fucoidan-alginate combined dressing group (2% FUC), and 5% fucoidan-alginate combined dressing group (5% FUC). The control group was treated with alginate dressing, in which the amount of fucoidan was 0%. Rats were fasted for 12h and anesthetized by intraperitoneal injection of 3% sodium pentobarbital (50 mg/kg), and then their back hair was removed using a shaving device. After the hair was removed, the skin on the backs of the rats was exposed, and a sterile pad was placed to position the rats in a prone position. The back of the rats was disinfected with 75% alcohol, and a sterilized square-type module with a side length of 1cm was taken as a marker at 0.5 cm to the side of the spine, 1 cm below the upper limb of the back of the rats. The marked area and surrounding skin were disinfected again, and then the full-thickness skin was removed along the marked position with sterile surgical scissors to form a rat skin wound model. According to the previous grouping, the rats in each group were applied with the corresponding dressings (2×2 cm square), which were fixed with medical tapes to prevent shedding. The nails of the rats were trimmed to prevent scratching and biting the dressings. Finally, the rats were returned to their respective cages and allowed to drink and eat freely. The day of successful modeling was the 0th day of trauma, and the dressings were changed at the same time as the 0th day from the next day, and the wound was photographed (26).

On days 3, 7, and 14 after trauma, five rats in the Control group, 2% FUC group, and 5% FUC group were randomly selected and anesthetized by intraperitoneal injection of 3% pentobarbital sodium (50 mg/kg). The skin and subcutaneous tissue within 5 mm of the wound were taken, and then the rats were sacrificed by spinal dislocation. Part of the wound tissue was fixed in a 4% paraformaldehyde fixative solution, and the other part was quickly put into liquid nitrogen for further assays.

### Assessment of the wound healing rate

The wound was photographed using a camera, and during the process, a ruler was placed under the wound to ensure consistent control over the angle and distance of each photograph. Image Pro Plus 6.0 software was used to measure the wound area and calculate the wound healing rate. The formula for calculating the wound healing rate is as follows: Wound healing rate (%) = (OA-UA)/OA×100% (OA refers to the original wound area, which is the wound area on day 0 of trauma. UA refers to the unhealed wound area, which was the wound area on the day of the photograph)(27).

### Histopathological analysis

Wound tissues were fixed by immersion in 4% paraformaldehyde and then embedded in paraffin. The tissues were cut into 5 μm-thick sections and stained with hematoxylin-eosin (H&E) solution. The sections are sealed with neutral gum, left overnight, and then observed and photographed with a light microscope (Ni-U, Nikon, Tokyo, Japan)(28). 

### White blood cell count

A total of 0.2 ml of blood was collected from the orbits of rats in each group on the 0, 3, 7, and 14 days after injury and placed into anticoagulant tubes containing EDTA. The white blood cell count of each sample at each time point was subsequently analyzed using an automatic blood cell analyzer and recorded (29).

### Enzyme-linked immunosorbent assay (ELISA)

Serum samples were collected from the rats on the 3rd, 7th, and 14th days after injury, and the levels of IL-1β (Cat. No.: PI303) and TNF-α (Cat. No.: PT516) were quantified using ELISA according to the manufacturer’s instructions. The levels of TGF-β1 (Cat. No.: PT878) were also measured(28).

### Masson’s trichrome staining

Collagen fiber deposition and distribution in wound tissues were detected by Masson’s trichrome staining experiment. The paraffin sections of wound tissues were stained with hematoxylin, ponceau, and aniline blue and finally sealed with neutral gum. After being left overnight, the sections are placed under a light microscope (Ni-U, Nikon, Tokyo, Japan), and five fields of view are randomly selected for photography. Using blue collagen fiber deposition as a positive signal, the ImageJ software was used to determine the wound collagen fiber deposition area and the total area of the wound in the field of view. The formula for calculating the collagen fiber deposition ratio is as follows: Collagen fiber deposition ratio (%) = CA/TA×100% (CA refers to collagen fiber deposition area, and TA refers to the total area of the wound in the field of view)(30).

### Immunohistochemistry

We incubated the endogenous peroxidase-blocking solution dropwise on the sections for 10 min at room temperature, then placed the sections in citric acid (PH 6.0) buffer and microwaved over medium heat for 5 min for antigen retrieval. The tissues on the sections were circled with an immunohistochemical pen and incubated at 37 ^°^C for 30 min after dropping 5% BSA. Then the sections are incubated overnight with primary antibodies (IL-1β, sc-52012, 1:200; TNF-α, sc-52746, 1:200; TGF-β1, sc-130348, 1:500) at 4 ^°^C and subsequently with secondary antibodies (ab150113, 1:5000) for 1 hr. Finally, the diaminophenylamine (DAB) chromogenic solution was added, and then the specimen was counterstained with hematoxylin. Immunohistochemistry was examined using a light microscope (Ni-U, Nikon, Tokyo, Japan) and analyzed with Image J software (30).

### Western blotting assay

The total protein of wound tissue was extracted using radioimmunoprecipitation assay (RIPA) and Phenylmethylsulfonyl fluoride (PMSF). Then, the concentration was determined by Pierce bicinchoninic acid (BCA) Protein Quantification Kit (Vazyme, Nanjing, China). Equal protein samples of 20 μg were subjected to 7-12% SDS-PAGE separation and subsequently transferred onto a PVDF membrane. After washed with TBST, the PVDF membrane was incubated with primary antibodies (β-actin, Cat. No.: 380624, 1:10000; α-SMA, Cat. No.: R380653, 1:1000; Col I , Cat. No.: R380760, 1:2000; Col III, Cat. No.: R23957, 1:1000; p-Smad/2/3 Cat. No.: AP1343, 1:1000) overnight at 4 ^°^C, followed by incubation with secondary antibodies (Cat. No.: ab6721, 1:10000) for 1 hr. Finally, the enhanced chemiluminescence (ECL) Buffer (Vazyme, Nanjing, China) was spread all over the PVDF membrane for visualization, and the gray level analysis of the obtained bands was performed by Image J software (31).

### Statistical analysis

GraphPad Prism 8.0 was used for statistical analysis (GraphPad, San Diego, CA, USA). The data are expressed as Mean±SD. The statistical differences between the groups were tested using one-way ANOVA. In all cases, *P*<0.05 was considered statistically significant. Each experiment was repeated three times.

## Results

### Effect of fucoidan dressing on wound healing

**Figure 1:**
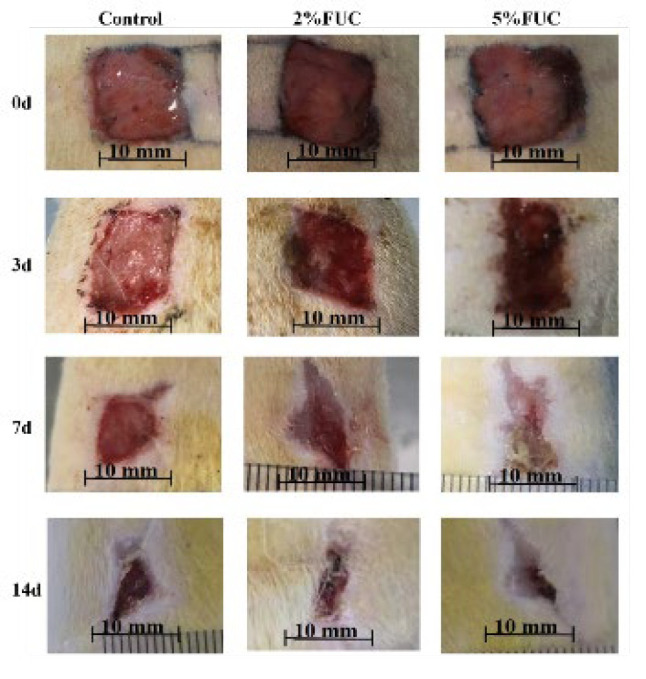
Healing status of wounds in rats with full-thickness skin defects in each group

The condition of the wounds in each group at 0, 3, 7, and 14 days post-injury is illustrated in Figure 1. On the day the wound model was established (Day 0), wounds in all groups exhibited congestion, redness, swelling, and serous exudation. By the third day post-injury, epithelialization was largely completed in all groups, and wound contraction was evident, with the most pronounced contraction observed in the 5% FUC group. The wounds in the 2% FUC and 5% FUC groups developed small, dark-red scabs, while scabs were not noticeable in the Control group, which showed minor inflammatory exudation. By the seventh day after injury, there was no significant exudation in any group. However, wounds in the 2% FUC and 5% FUC groups had visibly narrowed and contracted more distinctly compared to those in the Control group. The wound color in the Control group appeared red, likely due to inflammatory response and hyperemia. At 14 days post-injury, further healing was observed in all groups, with notably better outcomes in the 5% FUC group.

**Figure 2:**
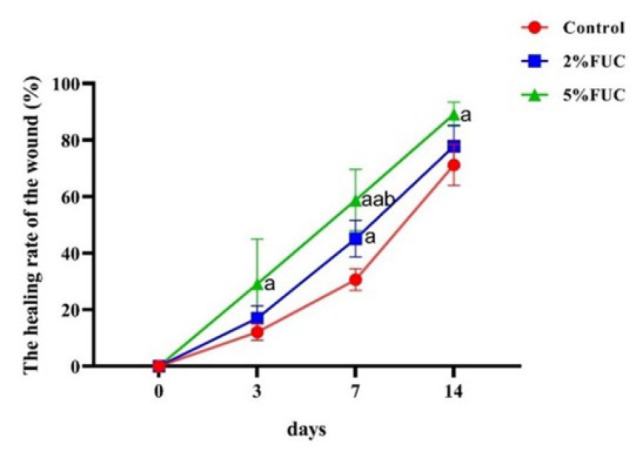
Wound healing rates of full - thickness skin - defect rats in each group at 3, 7, and 14 days after injury were analyzed

As illustrated in Figure 2, fucoidan dressings demonstrated the potential to accelerate wound healing in rats. Three days post-injury, the wound healing rate in the 5% FUC group was 2.4 times higher than that of the Control group (*P*<0.05). By day 7, the 5% FUC group continued to show a markedly improved wound healing rate, which was 1.9 times greater than that of the Control group (*P*<0.01) and 1.3 times greater than the 2% FUC group (*P*<0.05). Consistent with the results observed on days 3 and 7, the wound healing rate in the 5% FUC group remained significantly higher on day 14, reaching a value 1.3 times greater than that of the Control group (*P*<0.05). Conversely, no significant difference in wound healing rates was observed between the 2% FUC group and the Control group on days 3 and 14 post-injury (*P*>0.05). However, on day 7, the 2% FUC group displayed a significantly improved wound healing rate compared to the Control group (*P*<0.05). These results demonstrated that the 5% fucoidan dressing, when combined with alginate, significantly outperformed the alginate dressing alone in promoting wound healing across all measured time points. In contrast, the 2% fucoidan-alginate combined dressing showed benefits, but only at a single intermediate stage.

### Effect of fucoidan dressing on the pathological changes of wounds

**Figure 3:**
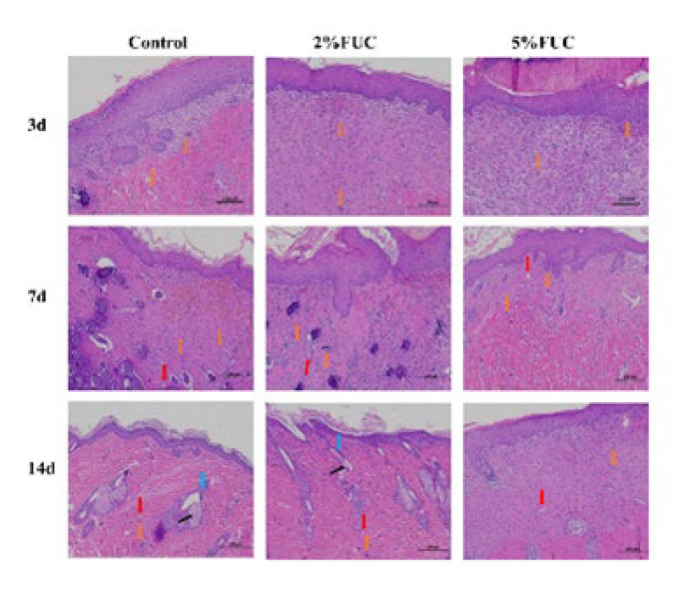
Representative photographs of H&E staining sections of wound tissue from full - thickness skin - defect rats at 3, 7, and 14 days after injury were taken

The H&E staining results of rat wounds in each group are presented in Figure 3, with observations made on days 3, 7, and 14 post-injury. On day 3, the wounds in all groups exhibited complete epidermal cell coverage, active dermal cell proliferation, and the initiation of collagen fiber production. In the 5% FUC group, fibroblast proliferation and granulation tissue formation were particularly pronounced. By day 7 post-injury, skin accessory glands and small blood vessels had developed in the dermis of the Control and 2% FUC groups, accompanied by notable inflammatory cell infiltration. In contrast, the 5% FUC group displayed fewer skin accessory glands and blood vessels in the dermis, along with milder inflammatory infiltration. Notably, collagen fibers in the 5% FUC group were more abundant and better aligned compared to the other two groups. By day 14, the Control and 2% FUC groups exhibited neatly developed hair follicles, whereas the 5% FUC group showed no significant development of skin accessory glands. Overall, the combination of fucoidan with alginate dressings, particularly at 5%, surpasses alginate dressings alone in promoting wound healing and regulating inflammation.

### Effects of fucoidan dressing on wound infection and levels of TGF-β1, IL-1β, and TNF-α

**Figure 4.**
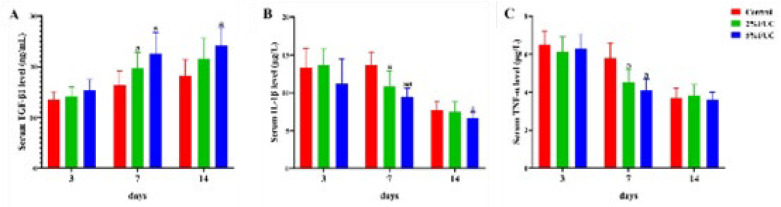
Effect of alginate fucoidan dressings on whole leukocyte counts of full - thickness skin - defect rats at 0, 3, 7, and 14 days after injury was analyzed

**Figure 5:**
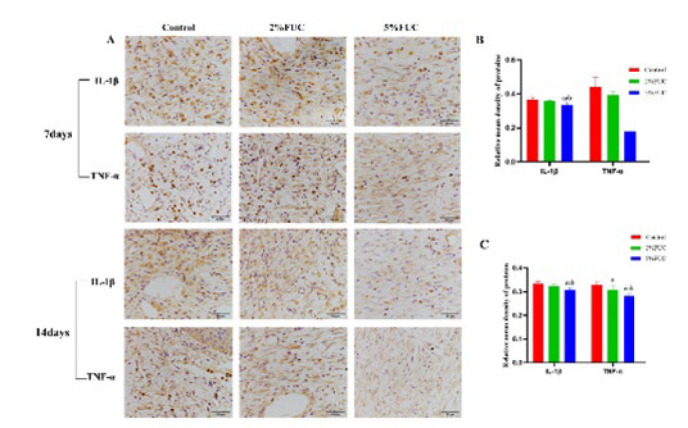
Effect of fucoidan dressing on TGF-β1, IL-1β and TNF-α levels in rats with full-thickness skin defect at 3, 7 and 14 days post-injury

It was shown that the number of white blood cells in the Control group was significantly higher than that in the fucoidan-treated group at each time point. Fucoidan treatment was found to reduce the number of white blood cells in a dose-dependent manner, thereby improving wound infection in rats. Furthermore, a significant increase in the number of white blood cells was observed on days 3 and 7. The combined fucoidan-alginate dressing exhibited a more pronounced effect in comparison to the alginate dressing alone (Figure 4). As shown in Figure 5, fucoidan-alginate combined dressings significantly increased TGF-β1 levels, reduced inflammatory factors IL-1β and TNF-α, and accelerated wound healing during the rapid healing phase.

### Effect of fucoidan dressing on inflammatory cytokines

**Figure 6:**
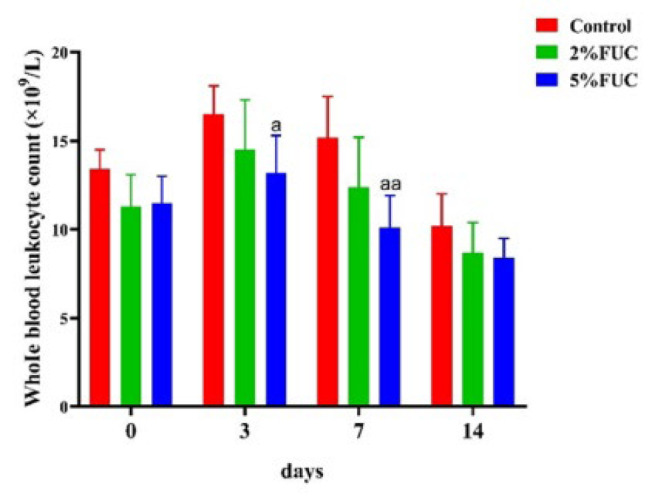
Effect of fucoidan dressing on inflammatory cytokines in full - thickness skin - defect rats at 7 and 14 days post - injury was studied

**Figure 7:**
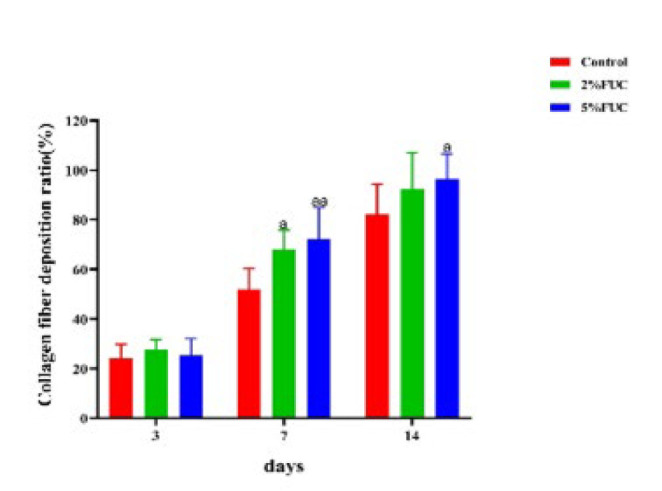
Representative photographs of Masson staining sections of wound tissue in rats with full-thickness skin defect at 3, 7 and 14 days post-injury

The inflammatory phase of wound healing comprises two key components: inflammatory cell infiltration and the release of inflammatory cytokines (32, 33). As illustrated in Figure 6, IHC analysis revealed that seven days after injury, TNF-α and IL-1β levels in the 5% FUC group were significantly lower compared to the Control group, showing reductions of 30.3% and 8.5%, respectively (*P*<0.01). Compared to the 2% fucoidan-alginate combined dressing, the 5% fucoidan-alginate combined dressing proved more effective in mitigating wound inflammation, with TNF-α and IL-1β levels decreased by 22.0% (*P*<0.01) and 6.4% (*P*<0.05), respectively. Results on day 14 post-injury (Figure 7) were consistent with those observed on day 7, showing significantly lower levels of TNF-α and IL-1β in wounds treated with the 5% group dressing compared to both the Control group (*P*<0.01) and the 2% FUC group (*P*<0.05). Additionally, a distinct reduction in TNF-α levels was observed in the 2% FUC group compared to the Control group on day 14, with a decrease of 6.1% (*P*<0.05). These findings indicate that incorporating fucoidan into alginate dressings effectively reduces wound inflammation, with this effect persisting through the later stages of wound healing.

### Effect of fucoidan dressing on collagen production in wounds

During wound healing, the formation of new tissue relies on collagen synthesis (34). The results of Masson staining performed on the wounds of each group at 3, 7, and 14 days post-injury are presented in Figure 7. On the third day after the injury, a small amount of collagen fiber was observed to be scattered within the newly formed tissues across all groups, with no significant differences identified among them. By the seventh day post-injury, the collagen fiber deposition ratio within the dermis of rats in each group had significantly increased. Notably, compared to the Control group, the collagen fibers in the 2% FUC group and the 5% FUC group exhibited a denser and more organized arrangement. By the 14th day after injury, the wound epithelium in all groups showed progressive proliferation and differentiation into squamous epithelium. Collagen distribution within the dermis became more uniform, forming abundant collagen fiber bundles. Among the groups, the 2% FUC and 5% FUC groups exhibited higher collagen maturity, more closely resembling the composition and structure of normal skin.

**Figure 8:**
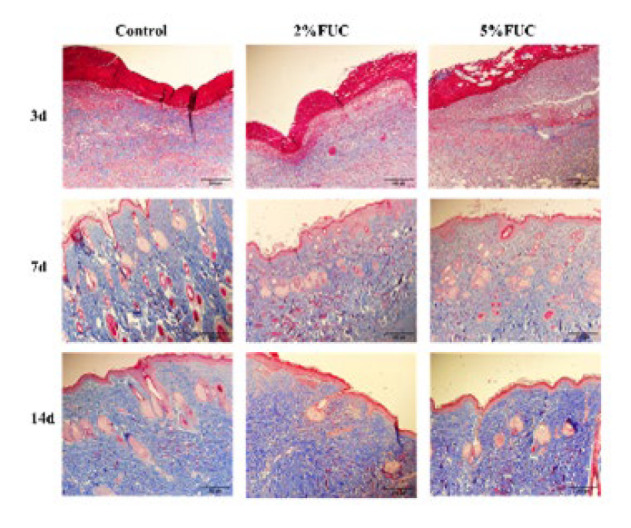
Collagen fiber deposition ratio in wounds of full-thickness skin-defect rats at 3,7, and 14 days post – injury

As illustrated in Figure 8, the collagen fiber deposition in rat wounds was assessed on days 3, 7, and 14 post-injury using Masson staining. While no significant differences in wound collagen fiber deposition were observed among the three groups on day 3 post-injury (*P*>0.05), the fucoidan dressing groups demonstrated higher collagen fiber levels compared to the Control group on days 7 and 14. On day 7, the collagen fiber deposition in the wounds of rats treated with the 5% FUC group and the 2% FUC group showed notable increases, reaching 1.4 times (*P*<0.01) and 1.3 times (*P*<0.05), respectively, compared to the Control group. By day 14, the wounds in the 5% FUC group displayed a significantly elevated collagen fiber deposition, measuring 1.2 times that of the Control group (*P*<0.05).

This finding suggested that, in comparison to alginate dressings used in isolation, fucoidan-alginate combined dressings—particularly those containing 5% fucoidan—significantly enhanced the collagen fiber content and maturity in wounds, thereby effectively promoting wound healing in rats.

### Effect of fucoidan dressing on TGF-β1/Smad signaling pathway

**Figure 9:**
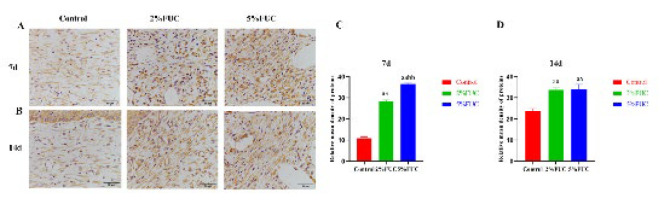
Effect of fucoidan dressing on TGF-β1 levels in rats with full-thickness skin defect

**Figure 10:**
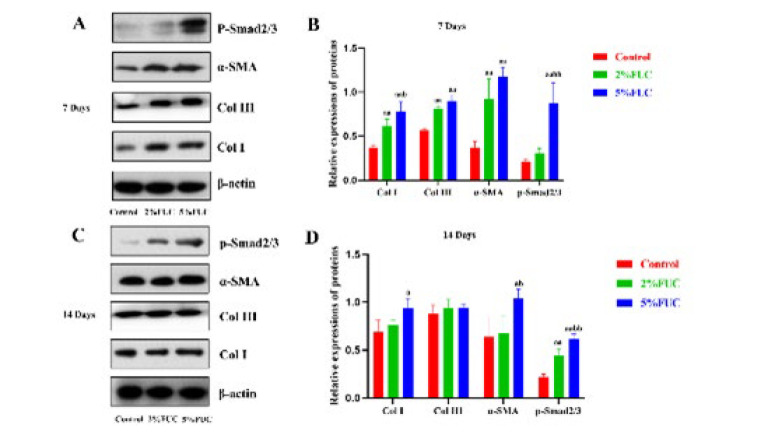
Expression of TGF - β1/Smad signaling pathway - related proteins in each group of rats with full - thickness skin defects after 7 days

TGF-β1 activates the TGF-β1/Smad signaling pathway, leading to the phosphorylation of the Smad2/3 complex (p-Smad2/3), which regulates α-SMA transcription and promotes myofibroblast differentiation (13, 15). Myofibroblasts, the primary cells responsible for collagen production, are commonly identified through the expression of α-SMA, a specific marker (35, 36). IHC analysis results (Figure 9) revealed that TGF-β1 levels in both the 5% FUC and 2% FUC groups were significantly elevated compared to the control group at both 7 and 14 days post-injury. As shown in Figure 10, western blot analysis of wound tissues indicated that, at 7 days after injury, rats treated with the 5% fucoidan-alginate combined dressing exhibited significantly increased expression levels of Col I, α-SMA, and p-Smad2/3, reaching 1.16-fold, 2.16-fold, and 3.24-fold compared to the control group, respectively (*P*<0.01). Similarly, the 2% FUC group showed elevated expression levels of Col I and α-SMA, with 0.70-fold and 1.48-fold increases relative to the control group (*P*<0.01). Western blot analysis performed on day 14 post-injury further demonstrated that the expression levels of Col I, α-SMA (*P*<0.05), and p-Smad2/3 (*P*<0.01) in the 5% FUC group remained significantly elevated, exceeding those observed in the control group. However, in the 2% FUC group, only the expression level of p-Smad2/3 showed a significant difference compared to the control group on day 14 post-injury (*P*<0.01). 

These findings suggest that fucoidan-alginate combined dressings enhance collagen production by upregulating the TGF-β1/Smad signaling pathway, thereby promoting wound healing.

## Discussion

In this study, we examined the impact of fucoidan-alginate combined dressing on wound healing in rats with full-thickness skin excisions. The results revealed that the application of fucoidan-alginate combined dressing notably accelerated wound healing, reduced wound inflammation, and promoted collagen synthesis in the rats.

Disturbances in wound-related cell behavior can result in healing disorders and the development of chronic, non-healing wounds, a condition whose prevalence has risen significantly worldwide in recent years (5). Excessive secretion of proinflammatory cytokines may prolong the inflammatory phase and exacerbate wound inflammation, ultimately leading to chronic wounds or hypertrophic scars (21, 37). Fucoidan dressings, by limiting the expression of IL-1β and TNF-α, have been shown to reduce wound inflammation (38). This anti-inflammatory effect persists through the later stages of wound healing, protecting the tissue from further damage and accelerating the overall healing process. TGF-β1 plays a crucial role in promoting extracellular matrix (ECM) synthesis and preventing its degradation via the TGFβ1/Smad signaling pathway (39, 40). Jin *et al*. demonstrated that IL-1β can disrupt the TGF-β1/Smad pathway by upregulating Smad7 and down-regulating TGF-RII receptors (14). Our study found that in the 5% FUC group, the levels of TGF-β1, Col I, Col III, α-SMA, and p-Smad2/3 observed after 7 and 14 days post-injury indicated that fucoidan-alginate combined dressings activate the TGF-β1/Smad signaling pathway, thereby enhancing collagen synthesis and facilitating wound contraction.

Fucoidan demonstrated anti-inflammatory effects by reducing neutrophil adhesion, limiting leukocyte recruitment, and downregulating pro-inflammatory factors in macrophages (41). Shi *et al*. (42) revealed that fucoidan possesses the potential to protect the intestinal mucosal barrier by suppressing inflammatory factors, such as IL-1β, and alleviating inflammatory responses. Similarly, Takahashi *et al*. (43) observed that a two-week course of fucoidan intake significantly decreased IL-1β and TNF-α levels in patients with advanced cancer, helping to counteract the detrimental effects of heightened inflammation on lifespan. Consistent with these findings, our study found that the use of fucoidan-alginate combined dressing produced comparable benefits in accelerating wound healing. In the 5% FUC group, IL-1β and TNF-α levels were markedly lower than those in the Control group at both 7 and 14 days post-injury. These results suggest that fucoidan-alginate combined dressings may facilitate wound healing by mitigating inflammation through the inhibition of IL-1β and TNF-α expression.

Type I and Type III collagens are the predominant components of the extracellular matrix (ECM) and are extensively distributed across both skin and tendon tissues(44). During the initial phase of wound healing, Type III collagen is primarily synthesized. As the healing process progresses, Type III collagen gradually decreases and is replaced by Type I collagen, thereby enhancing the tensile strength of the scar tissue (5, 45). An impaired TGF-β1/Smad signaling pathway has been identified as one of the mechanisms contributing to diminished collagen synthesis(14). Consequently, the up-regulation of the TGF-β1/Smad signaling pathway has been shown to promote collagen production. The process begins when activated TGF-β1 binds to the TGF-βRII receptor on the cell surface. This interaction facilitates the recruitment and binding of TGF-βRI, subsequently forming heterotetrameric complexes between these two receptors (14). This binding event triggers the phosphorylation of TGF-βRI, which then phosphorylates Smad2 and Smad3 (R-Smads). The phosphorylated Smads (p-Smad2/3) translocate to the nucleus in conjunction with Smad4 (co-Smad), where they bind to the Smad binding element (SBE)(46, 47). The binding of p-Smad2/3 to the SBE regulates the transcription of α-SMA and increases its expression, ultimately inducing the transformation of fibroblasts into myofibroblasts (13, 15, 48). α-SMA is widely recognized as a specific marker for assessing myofibroblast generation. These myofibroblasts are the primary source of collagen production (35, 36, 49).

Previous research has shown that fucoidan effectively inhibits the activation of the TGF-β1 signaling pathway in various cell lines, including those of liver cancer, breast cancer, and non-small cell lung cancer, thereby suppressing the metastasis of malignant cells (20). In our study, the application of fucoidan-alginate combined dressings was found to promote the activation of the TGF-β1/Smad signaling pathway during wound healing, leading to an increase in α-SMA expression and ultimately accelerating wound contraction. Moreover, the absence of observable skin gland development in the wounds of rats treated with 5% fucoidan-alginate combined dressings may stem from an imbalance between the synthesis rate of extracellular matrix (ECM) components, such as collagen, and the regeneration rate of skin-associated glands, including hair follicles and sweat glands. This imbalance creates a barrier within the wound, obstructing the outward growth of these glands through the dermis (50, 51).

Full-thickness skin was excised from the dorsal region of each rat to create a wound, and the corresponding dressing was replaced daily in every group of rats. Representative photographs were taken at 0, 3, 7, and 14 days after injury

FUC: Fucoidan

Each value represents the mean±SD (n=5/group). a, compared with the control group, *P<*0.05; aa, compared with the control group, *P<*0.01; b, compared with the 2% FUC group, *P<*0.05

FUC: Fucoidan

Yellow arrows for inflammatory cell infiltration, blue arrows for hair follicles, black arrows for sebaceous glands, and red arrows for small blood vessels. Seven days after injury, skin accessory glands and small vessels were formed in the dermis of the Control group and 2% FUC group, and the infiltration of inflammatory cells was relatively visible. In the 5% FUC group, there were fewer skin accessory glands and blood vessels in the dermis of the wound, and the inflammatory infiltration was mild (scale bar=200 µm)

FUC: Fucoidan; H&E: Hematoxylin and Eosin

Each value represents the mean±SD (n=5/group). a, compared with the control group, *P<*0.05; aa, compared with the control group, *P<*0.01

FUC: Fucoidan

Each value represents the mean±SD (n=5/group). a, compared with the control group, *P<*0.05

FUC: Fucoidan; TGF-β1: Transforming growth factor-β1; IL-1β: Interleukin-1beta; TNF-α: Tumor necrosis factor-α

(A) Results of IHC staining of wounds of rats in each group (scale bar=50 µm). (B-C) The mean density of IL-1β and TNF-α in wounds of rats in each group. Each value represents the mean±SD (n=5/group). a, compared with the control group, *P<*0.05; aa, compared with the control group, *P<*0.01; b, compared with the 2% FUC group, *P<*0.05; bb, compared with the 2% FUC group, *P<*0.01

FUC: Fucoidan; IL-1β: Interleukin-1beta; TNF-α: Tumor necrosis factor-α

A large number of collagen fiber bundles were formed in the wound tissue 14 days after injury. The collagen in the 2% FUC group and 5% FUC group was denser and more mature (scale bar=200 µm)

FUC: Fucoidan

Each value represents the mean±SD (n=5/group). a, compared with the control group, *P<*0.05; aa, compared with the control group, *P<*0.01

FUC: Fucoidan

(A) Results of IHC staining of wounds of rats in each group on day 7 of injury (scale bar=50 µm). (B) Results of IHC staining of wounds of rats in each group on day 14 of injury (scale bar=50 µm). (C) The mean density of TGF-β1 in wounds of rats in each group on day 7 of injury. (D) The mean density of TGF-β1 in wounds of rats in each group on day 14 of injury. Each value represents the mean±SD (n=5/group). a, compared with the control group, *P<*0.05; aa, compared with the control group, *P<*0.01; b, compared with the 2% FUC group, *P<*0.05

FUC: Fucoidan; TGF-β1: Transforming growth factor-β1

Each value represents the mean±SD (n=5/group). a, compared with the control group, *P<*0.05; aa, compared with the control group, *P<*0.01; b, compared with 2% fucoidan (FUC) group, *P<*0.05; bb, compared with 2% FUC group, *P<*0.01

## Conclusion

In conclusion, our findings suggest that alginate dressings enriched with fucoidan can promote wound healing in SD rats by reducing IL-1β and TNF-α levels to mitigate inflammation and enhancing the transcription of α-SMA to facilitate collagen synthesis through the TGF-1/Smad pathway. While this approach may help prevent the development of chronic wounds, it has been noted that dressings containing 5% fucoidan could hinder the regeneration of skin accessory glands. Therefore, further studies are necessary to determine the optimal fucoidan concentration for therapeutic use.

## Data Availability

The data used to support the findings of this study are available from the corresponding author upon request.

## Declaration

We have not used any AI tools or technologies to prepare this manuscript.
